# Evaluation of healthcare professionals' understanding of fluoroquinolones' safety profile, usage, and boxed warnings in Pakistan

**DOI:** 10.1186/s40545-023-00674-6

**Published:** 2023-11-27

**Authors:** Muhammad Abbas, Kashif Kashmiri, Inayat Ur Rehman, Zahid Ali, Aziz Ur Rahman, Asad Khalil, Long Chiau Ming, Muhammad Shafique, Tahir Mehmood Khan

**Affiliations:** 1 Department of Pharmacy, Abdul Wali Khan University Mardan, Mardan, Pakistan; 2 Department of Pharmacy, University of Peshawar, Peshawar, Pakistan; 3 Department of Urology, North West General Hospital and Research Center, Peshawar, Pakistan; 4 Department of Medicine, Lady Reading Hospital, Peshawar, Pakistan; 5 School of Medical and Life Sciences, Sunway University, Jalan Universiti, Bandar Sunway, Malaysia; 6 Department of Pharmaceutical Sciences, College of Pharmacy, Shaqra University, Shaqra, Saudi Arabia; 7 Institute of Pharmaceutical Sciences, University of Veterinary and Animal Sciences, Lahore, Pakistan

**Keywords:** Health care professionals, Fluoroquinolones, Box warning, Pakistan

## Abstract

**Introduction:**

Fluoroquinolones (FQs) is a distinct class of antibiotics which are prescribed and used quite frequently worldwide, despite the box warnings (BW) issued by Food and Drug Administration (FDA). Literature has shown in spite of BWs related to FQs there is minimal impact on health care professionals (HCPs) prescribing habits, potentially attributing towards limited and insufficient awareness. In Pakistan, FQs are mostly prescribed antibiotics for microbial treatments, therefore the purpose of this study was to determine the level of knowledge about the safety profile, use, and BW of FQs among HCPs working in Pakistan.

**Methods:**

A cross-sectional study was undertaken among the HCPs of Khyber Pakhtunkhwa province of Pakistan from October 2022 to December 2022. A validated questionnaire was used to assess the knowledge of HCPs regarding FQs, its safety profile and BW. A random convenient sample technique was used while recruiting HCPs in this study. As the HCPs comprised physicians, dentists, pharmacist and nurses, all were approached in person and the study objective was fully elaborated and explained to them. The statistic test like: one-way ANOVA, independent-*t* test, multivariate logistic regression were used keeping the *p*-value < 0.05 as statistically significant.

**Results:**

A total of *n* = 250 HCPs were approached, of which *n* = 186 HCPs completed the questionnaire with a response rate of 74.4%. FQs prescribing pattern was only assessed among the prescribers, i.e., physicians and dentists (39/186). The mean knowledge score for indications was 5.29 ± 3.05, while for the adverse effects was 7.70 ± 2.61. The highest score for knowledge for indications and adverse effect score was achieved by physicians followed by dentist. The mean knowledge score for the BW was 3.46 ± 2.93 and among the HCPs for the BW of FQs, 20.4% of the HCPs had appropriate knowledge score (score ≥ 50%). The knowledge score was significantly higher in males (*p* = 0.039), dentists (*p* = 0.001), HCPs having master/specialization level of education (*p* = 0.003), HCPs working in government sector hospitals (*p* = 0.010) and secondary care hospitals (*p* = 0.001) while the multivariate logistic regression analysis showed that HCPs working in primary care hospital (OR: 6.2) and secondary care hospital (OR: 20.3) were associated with the tendency to achieve 50% or above knowledge score.

**Conclusion:**

Findings of this study reveals the unsatisfactory knowledge of HCPs regarding the safety profile, use, and BW of FQs putting patients at heightened risks of FQs associated AEs. Therefore, it is crucial to implement a national antimicrobial stewardship program, seminars and lectures aimed at continuously updating the knowledge of HCPs, regardless of their specialties, and effectively restrict the misuse of antimicrobial and disseminate FDA BWs in clinical practice.

## Introduction

Antimicrobials, including antibiotics, are frequently prescribed in both inpatient and outpatient settings, with approximately half of all hospitalized patients receiving them for conditions such as pneumonia and urinary tract infections [1, 2]. In around 20% of the in-patients who are prescribed antibiotics experience adverse events (AEs). In addition, lack of drug-related knowledge significantly increases the risk of medication errors [3].

Fluoroquinolones (FQs) are among the fourth most widely used class of antibiotics [4], however they have been reported to cause serious and potentially permanent AEs of tendons, central nervous system (CNS), muscles, joints and nerves [5–7]. In United States, around 70% of FQs spending occurs in community pharmacies [8]. Similarly, 25% prescriptions of FQs are either unnecessary or not the first-line recommended therapy in outpatients [9]. In response to this, the FDA since 2008 issued multiple boxed warnings (BWs) regarding FQs, thereby limited their use based on the risk–benefit ratio [10]. Similarly, other BWs issued against FQs include worsening of myasthenia gravis in 2011, peripheral neuropathy (irreversible) in 2013, worsening of mental health and risk of hypoglycemic coma and aortic aneurysm in 2018, respectively [5, 6, 10]. According to the FDA adverse event reporting system (FAERS) database, till May 2023 tendon rupture is the most frequently reported AE with Levofloxacin (26.7%) and ciprofloxacin (17.6%) [4]. A study reported that FQs-related AEs result in more emergency department visits than cephalosporins, and macrolides [11]. Hence, the current guidelines recommend that FQs can be used as first-line drugs only if the benefits outweigh the risks [12].

FQs, despite considerable number of evidence regarding toxicities and numerous BWs, are still irrationally prescribed in various health settings [12]. While, following the issuance of BWs, few studies in the United States have shown decline in the FQs prescription [13, 14]. However, in many other countries around the world, these warning have not had a significant impact on the prescribing patterns of FQs [6, 12, 15–17]. Violating BWs can be dangerous for patients in terms of developing serious AEs. It is reported that approximately 7 out of 1000 outpatients receive prescriptions of drugs with BW violations, thereby putting them at greater risk of developing severe AEs [18].

In terms of antibiotic use, Pakistan ranks third among low- and middle-income nations, with a 65% increase in daily defined doses (DDDs) seen between 2000 and 2015 [19]. The report also showed that the consumption of FQs has significantly increased, with nearly 208,000 more people being prescribed FQs per day [19]. In Pakistan, FQs are prescribed mostly to patients with GIT infections (bacterial origin) and are the most frequently prescribed antimicrobials with a prescription percentage of 19.4% in primary health care centers and 22.8% in basic health units [20]. In addition, junior doctors at tertiary care hospitals tend to follow the prescriptions of senior or specialist doctors, which accounts for the higher prescription of FQs [21, 22].

The excessive consumption and irrational prescribing of FQs by HCPs may be linked to insufficient of knowledge and awareness regarding FQs-related BWs, safety profiles, and prescribing guidelines. It is crucial to assess HCPs understanding and knowledge of FQs in order to create and implement targeted education interventions and ensure that these medications are appropriately used. Therefore, this study aimed to assess physicians', pharmacists, dentists', and nurses' awareness and degree of knowledge on the safety profiling, its associated BWs, and the use of FQs. The results of this study will aid in the development of training and educational programs aimed at providing an ongoing educational activities for the HCPs. Furthermore, the findings will also assist in reducing and limiting the irrational usage of these FQs keeping in mind the BWs issued by FDA.

## Method

### Study design and setting

A cross-sectional study was performed from October 2022 to December 2022 among the HCP’s employed in health care settings of Khyber Pakhtunkhwa province of Pakistan.

### Population

The HCP’s including physicians, dentists, pharmacist and nurses working in government and private health care setting were recruited. HCPs with at least one year of experience who were willing to participate in the study were included in the study; whereas, those unwilling to participate or had less than one year of experience were excluded. As part of the ethics approval process, informed written consent was obtained from the HCPs.

### Ethics approval

The ethics committee of Abdul Wali Khan University Mardan granted ethics approval (EC/AWKUM/2022/24/479 (dated 26/06/2022).

### Study questionnaire

In order to carry out this research on the knowledge of HCP’s, practice toward BW of FQs, a validated questionnaire previously used in the study was selected [10]. The questionnaire comprised 62 items/questions having five sections. The section-1 comprised 8 questions related to demographics characteristics including gender, age, field of specialty, level of education, working status (government or private), level of education, working place and experience. Section-2 comprised 13 names of drugs belonging to the FQs class, which were intended to be filled by practicing and prescribing HCP’s (physicians and dentists). The participants were requested to provide their responses on a four item scale which comprised options like “no use”, “frequent use”, “less frequent use”, and “new drug”. Additionally this section comprised question related to the mechanism of action of FQs, the correct answer (DNA gyrase/topoisomerase IV inhibitor) was assigned with a score of "1" and the incorrect with a score of “0". Section-3 consists of 12 questions regarding FQs indications, HCPs were instructed to respond to any of the following options, i.e., “Yes”, “No” and “Not sure”. The option “Yes” was assigned score of 1 and for “No” and “Not sure” with score of 0. Similarly, another section comprised 18 adverse effects associated with FQs use, having options “Yes”, “No” and “Not sure”. The HCPs were instructed to respond to these adverse effects associated with use of FQs on these options, the option “Yes” was assigned score of 1 and for “No” and “Not sure” with score of 0. The last section of the questionnaire comprised nine questions related to BW issued by FDA in 2018 to evaluate the knowledge and awareness score of the HCP’s toward the BW. In connection to these nine questions for BW, question no 1 in this section as related to “Do you know that FDA has communicated the most recent black box warnings regarding the use of FQs in 2018? These warnings include new side effects/adverse events of these drugs” and the HCP’s had to either to select options “Yes” or “No” for each of the BW. The option “Yes” was scored as 1 while for option “No” as 0; while the rest eight questions in this section was related to eight list of BWs and the HCPs had to select any of the available options, i.e., “Yes”, “No” and “Not sure”. As these BWs were announced by the FDA from 2008–2018 keeping in mind this the option “Yes” was scored 1 while other options were scored 0. This study questionnaire included 40 items having a total knowledge score of 40. The knowledge score of each section was also calculated keeping in mind the above stated scoring guide. Considering the complexity level of the study tool, a knowledge score of 50% of HCPs was categorized as appropriate knowledge score and was used as a reference for comparison in this study [10].

### Sample size

A random convenient sample technique was used while recruiting HCPs in this study. As the HCPs comprised physicians, dentists, pharmacist and nurses, all were approached in person and the study objective was fully elaborated and explained to them, and then a written informed consent was taken from them if they agreed to participate in the study. After completing the questionnaire, it was reviewed for completeness before being subjected to analyses.

### Data analysis

The data were analyzed by using Statistical Package for the Social Sciences (SPSS version-22). The findings for categorical variables in the study were reported as frequency and percentages, while the findings for continuous variables were presented as mean and standard deviations. The mean knowledge score of the HCPs was compared while using one-way ANOVA and independent-*t* test, where appropriate for the gender, age, field of specialty, level of education, working status (government or private), level of education, working place and experience and also checked for post hoc Tukey analysis for specific knowledge score of the HCPs, which deemed appropriate. A binary logistic regression was performed to identify the factors/predictors independently associated with the knowledge score of the HCPs. In order to do so, the knowledge score of HCPs were categorized into two categories, i.e., knowledge score < 50% and knowledge score > 50%. Univariate logistic regression was performed and those factors having *p*-value < 0.25 [23] were subjected to multivariate logistic regression. The findings of the logistic regression were expressed in odds ratio (OR) and 95% confidence intervals while the *p*-value < 0.05 was considered as statistically significant.

## Results

A total of *n* = 250 health care professional (HCPs) was approached in this study, of which *n* = 186 HCPs completed the questionnaire (response rate: 74.4%). Among the participated HCPs majority 57.5% were females, while 67.7% of the HCPs were from the age group of 18–30 years and 53.8% were pharmacist. Regarding level of education, 75.8% were having graduate level of education; while, 54.3% were working in government sector. Regarding working place and professional experience, 39.8% were working in tertiary care hospitals and 68.3% having less than 5 years of experience as shown in Table 1.

**Table 1:** Demographic characteristics of HCPs

	Frequency	Percent
*Gender*		
Male	79	42.5
Female	107	57.5
*Age*		
18–30 years	126	67.7
31–40 years	57	30.6
41 years and above	3	1.6
*Field of specialty*		
Physician	25	13.4
Dentist	13	7.0
Pharmacist	100	53.8
Nurses	48	25.8
*Level of education*		
Graduation	141	75.8
Master/specialization	45	24.2
*Working status*		
Government	101	54.3
Private	85	45.7
*Working place*		
Private hospital	44	23.7
Primary care hospital	14	7.5
Secondary care hospital	13	7.0
Tertiary care hospital	74	39.8
Community pharmacy	17	9.1
Hospital pharmacy	20	10.8
Others	4	2.2
*Experience*		
Less than 5 years	127	68.3
5–10 years	34	18.3
More than 10 years	23	12.4

FQs prescribing pattern was only assessed among the prescribers, i.e., physicians and dentists (39/186). Among a list of antibiotics prescribed by the physicians and dentists, nalidixic acid (52.6%), lomefloxacin and ofloxacin (42.1%), respectively, and gemifloxacin (39.5%) were the most frequently prescribed FQs; while, 52.6% of the prescribers reported gatifloxacin (52.6%), trovafloxacin (36.8%) and sparfloxacin (36.8%) are new drugs shown in Table 2.

**Table 2:** Physicians and dentists prescribing pattern regarding FQs

	I do not prescribe this drug		I prescribe this drug frequently		I prescribe this drug less frequently		This drug is new for me	
	*N*	%	*N*	%	*N*	%	*N*	%
Ciprofloxacin	0	0	12	31.6	26	68.4	0	0
Levofloxacin	0	0	13	34.2	25	65.8	0	0
Ofloxacin	4	10.5	16	42.1	15	39.5	3	7.9
Moxifloxacin	4	10.5	12	31.6	18	47.4	4	10.5
Gemifloxacin	4	10.5	15	39.5	7	18.4	12	31.6
Delafloxacin	9	23.7	16	42.1	4	10.5	9	23.7
Norfloxacin	14	36.8	11	28.9	2	5.3	11	28.9
Gatifloxacin	4	10.5	14	36.8	0	0	20	52.6
Lomefloxacin	8	21.1	16	42.1	2	5.3	12	31.6
Nalidixic acid	8	21.1	20	52.6	7	18.4	3	7.9
Cinoxacin	14	36.8	12	31.6	0	0	12	31.6
Trovafloxacin	10	26.3	11	28.9	3	7.9	14	36.8
Sparfloxacin	8	21.1	13	34.2	3	7.9	14	36.8

The mean knowledge score for indications was 5.29 ± 3.05, while for the adverse effects was 7.70 ± 2.61 (Table 3). The highest score for knowledge score for indications was achieved by physicians followed by dentists. Inter-professional comparisons showed that dentists score was significantly higher than pharmacist and nurses (*p* < 0.001 and *p* = 0.014), respectively (Table 4). The most commonly reported indications were urinary tract infections (61.8%), respiratory tract infections (61.3%) and otitis externa (50.0%) (Table 3). Majority of the HCPs (66.1%) reported that FQs are not indicated for bacterial eye infection, followed by corneal ulcer (49.5%).

**Table 3:** HCPs knowledge toward indications and adverse effects of FQs

	Yes		No		Not sure		Mean ± SD
	*N*	*%*	*N*	*%*	*N*	*%*	
*Indications*							
Bacterial eye infection	53	28.5	123	66.1	10	5.4	0.39 ± 0.59
Corneal ulcer	78	41.9	92	49.5	16	8.6	0.59 ± 0.64
Otitis externa	93	50.0	84	45.2	9	4.8	0.60 ± 0.58
Respiratory tract infections	114	61.3	57	30.6	15	8.1	0.77 ± 0.58
Urinary tract infections	115	61.8	38	20.4	33	17.7	0.97 ± 0.61
Skin & soft tissue infections	84	45.2	61	32.8	41	22	0.89 ± 0.73
Gonorrhea	58	31.2	83	44.6	45	24.2	0.80 ± 0.80
Surgical prophylaxis	56	30.1	91	48.9	39	21	0.72 ± 0.79
Anthrax	48	25.8	86	46.2	52	28	0.82 ± 0.84
Meningococcal meningitis	82	44.1	68	36.6	36	19.4	0.83 ± 0.73
Fistulating Crohn’s disease	66	35.5	91	48.9	29	15.6	0.67 ± 0.73
Typhoid fever	108	58.1	62	33.3	16	8.6	0.75 ± 0.60
Average knowledge score for indications domain (out of 12)							5.13 ± 1.59
*Adverse effects*							
Nauseas or vomiting	59	31.7	106	57	21	11.3	0.54 ± 0.69
Diarrhea	93	50	84	45.2	9	4.8	0.60 ± 0.58
Arthropathy	100	53.8	79	42.5	7	3.8	0.61 ± 0.56
Loss of appetite	88	47.3	75	40.3	23	12.4	0.72 ± 0.67
Musculoskeletal pain	82	44.1	78	41.9	26	14	0.72 ± 0.69
Renal impairment	88	47.3	77	41.4	21	11.3	0.70 ± 0.66
Hepatic impairment	83	44.6	77	41.4	26	14	0.73 ± 0.69
Headache	75	40.3	86	46.2	25	13.4	0.67 ± 0.70
Dizziness	59	31.7	96	51.6	31	16.7	0.65 ± 0.75
Seizures	61	32.8	86	46.2	39	21	0.75 ± 0.78
Dyspnea	81	43.5	83	44.6	22	11.8	0.67 ± 0.68
Trouble sleeping	82	44.1	69	37.1	35	18.8	0.82 ± 0.72
Altered smell sensation	73	39.2	73	39.2	40	21.5	0.82 ± 0.76
Asthenia	69	37.1	95	51.1	22	11.8	0.61 ± 0.69
Sensation abnormalities (peripheral neuropathy)	82	44.1	80	43	24	12.9	0.70 ± 0.69
Clostridium difficile infections	58	31.2	117	62.9	11	5.9	0.43 ± 0.60
Cardiovascular problems	88	47.3	86	46.2	12	6.5	0.60 ± 0.60
Photosensitivity or skin reactions	112	60.2	61	32.8	13	7	0.74 ± 0.58
Average knowledge score for adverse effects domain (out of 18)							7.70 ± 2.61

**Table 4:** Comparison of knowledge scores for indications and adverse effects among HCPs of various specialties

Specialty of HCPs	Knowledge score	*P*-value (vs. physicians)	*P*-value (vs. dentists)	*P*-value (vs. pharmacists)	*P*-value (vs. nurses)
*Indication score*					
Physician	5.52 ± 2.044	Reference	0.076	0.092	0.958
Dentist	6.77 ± 1.092	0.076	Reference	< 0.001*	0.014*
Pharmacist	4.73 ± 1.613	0.092	< 0.001*	Reference	0.106
Nurses	5.33 ± 0.930	0.958	0.014*	0.106	Reference
*Adverse effect score*					
Physician	9.8 ± 3.069	Reference	0.666	< 0.001*	0.004*
Dentist	8.85 ± 0.899	0.666	Reference	0.059	0.465
Pharmacist	7.02 ± 2.609	< 0.001*	0.059	Reference	0.355
Nurses	7.73 ± 1.976	0.004*	0.465	0.355	Reference

*P-value < 0.05 statistically significant, post hoc Tukey was used

Regarding the comparison of HCPs for the adverse effect score, the highest score was achieved by physicians followed by dentists. The inter-professional comparison for the adverse effects score showed the physician score was significantly higher than pharmacist (*p* < 0.001) and nurses (*p* = 0.004) as shown in Table 4. The most common adverse effects reported were photosensitivity or skin reactions (60.2%) and arthropathy (53.8%); while, 62.9% of the HCPs were unaware that clostridium difficile infections, dizziness (51.6%) and asthenia (51.1%) were the other common adverse effects of FQs (shown in Table 3).

The knowledge score of HCPs regarding the BW for FQs, was assessed by eight FDA BWs issued in 2018. Among BWs issued, majority of the HCPs recognized mental health side effects (54.3%), followed by hypoglycemia (49.5%) and disabling side effects of the tendons, muscles, nerves and peripheral neuropathy (45.2%) as the most common BW for FQs (shown in Table 5). The mean knowledge score for the BW was 3.46 ± 2.93.

**Table 5:** HCPs knowledge toward BW on FQs

	Yes		No		Not sure	
	*N*	%	*N*	%	*N*	%
Worsening of pre-existing myasthenia gravis	44	23.7	75	40.3	67	36
Disabling side effects of the tendons, muscles, nerves and joints	77	41.4	47	25.3	62	33.3
Hypoglycemia	92	49.5	36	19.4	58	31.2
Tendinitis or tendon rupturing	76	40.9	34	18.3	76	40.9
Restricted use of fluoroquinolones for certain uncomplicated infections	57	30.6	25	13.4	104	55.9
Aortic aneurysm/raptures or tears in aorta	50	26.9	19	10.2	117	62.9
Mental health side effects	101	54.3	27	14.5	58	31.2
Peripheral neuropathy	84	45.2	26	14	76	40.9
Average knowledge score for BW domain (out of 8)	3.46 ± 2.93 (Mean ± SD)					

Among the HCPs for the BW of FQs, 20.4% of the HCPs had appropriate knowledge score (score ≥ 50%). The knowledge score was significantly higher in males (*p* = 0.039), dentists (*p* = 0.001), HCPs having master/specialization level of education (*p* = 0.003), HCPs working in government sector hospitals (*p* = 0.010) and secondary care hospitals (*p* = 0.001) as shown in Table 6. However, knowledge score did not differ significantly among the age of HCPs and working experience. However, the multivariate logistic regression analysis showed that HCPs working in primary care hospital (OR: 6.2) and secondary care hospital (OR: 20.3) were associated with the tendency to achieve 50% or above knowledge score (shown in Table 7).

**Table 6:** Association of HCPS’s demographics with knowledge score

Variable	Mean ± SD	*p*-value
*Gender*		
Male	17.67 ± 6.10	0.039^*,a^
Female	16.42 ± 4.61	
*Age*		
18–30 years	17.08 ± 5.67	0.828^b^
31–40 years	16.60 ± 4.61	
41 years and above	17.67 ± 1.16	
*Field of education*		
Physician	24.08 ± 4.98	< 0.001^*,b^
Dentist	24.38 ± 1.39	
Pharmacist	15.09 ± 4.12	
Nurses	15.06 ± 2.96	
*Level of education*		
Graduation	16.77 ± 5.43	0.003^*,a^
Master/specialization	17.47 ± 4.94	
*Working status*		
Government	17.87 ± 5.48	0.010^*,a^
Private	15.84 ± 4.39	
*Working place*		
Private hospital	16.20 ± 4.34	< 0.001^*,b^
Primary care hospital	17.93 ± 6.52	
Secondary care hospital	24.00 ± 7.29	
Tertiary care hospital	16.82 ± 5.06	
Community pharmacy	15.18 ± 4.25	
Hospital pharmacy	14.30 ± 1.66	
Others	21.50 ± 4.04	
*Experience*		
Less than 5 years	17.03 ± 5.58	0.052^b^
5–10 years	15.12 ± 3.14	
More than 10 years	18.43 ± 5.53	

^a^Independent *t*-test; ^b^one-way ANOVA; *statistically significant *p*-value < 0.05

**Table 7:** Predictors of achieving ≥ 50% knowledge score

	Univariate binary logistic regression				Multivariate binary logistic regression			
	OR	CI 95%		*p*-value	OR	CI 95%		*p*-value
*Gender*								
Male	1.00 (Reference)							
Female	0.460	0.023	0.936	0.032*	–	–	–	–
*Age*								
18–30 years	1.00 (Reference)							
31 and above years	0.661	0.213	2.051	0.474	–	–	–	–
*Level of education*								
Graduation	1.00 (Reference)							
Master/specialization	1.057	0.470	2.377	0.893	–	–	–	–
*Working status*								
Government	1.00 (Reference)							
Private	0.369	0.172	0.794	0.011*	–	–	–	–
*Working place*								
Private hospital	1.00 (Reference)							
Primary care hospital	5.850	1.429	23.954	0.014*	6.183	1.331	28.716	0.020*
Secondary care hospital	17.550	3.911	78.758	0.000*	20.341	1.860	222.451	0.014*
Tertiary care hospital	1.983	0.667	5.898	0.218	0.873	0.116	6.552	0.895
Community pharmacy	1.671	0.353	7.924	0.518	1.408	0.285	6.945	0.674
Hospital pharmacy	0.000	0.000	–	0.998	0.000	0.000	–	0.998
Others	7.800	0.891	68.304	0.064	0.000	0.000	–	0.999
*Experience*								
Less than 5 years	1.00 (Reference)							
5–10 years	0.221	0.050	0.979	0.047*	0.078	0.012	0.510	0.008*
More than 10 years	1.886	0.726	4.901	0.193	2.246	0.742	6.800	0.152

Multivariate logistic regression was used, *statistically significant *p* < 0.05; *OR* odds ratio, *CI* confidence interval

## Discussion

To the best of our knowledge this is the first kind of its kind study performed among HCPs to evaluate their understanding of FQs, safety profile, usage, and BWs in Pakistan. FQs are among the fourth most common antibiotics that are most widely prescribed in the United States [4, 24]. In 2008 and 2013, the FDA issued BWs to inform the HCPs for taking cautions while making decision for the use of FQs that were associated with increased risk of tendinitis, neuropathy, hypoglycemia, psychiatric side effects, and possible aortic vessel rupture [13]. The findings of a recent meta-analysis revealed that the FQs user have 2.5 times higher risk of Achilles tendon rupture, and twofold higher risk of any tendon disorder than non-users [25]. Similarly, FQs users are twice more likely to suffer from peripheral neuropathy [26]. Despite the BWs and cautions, recent data indicate that BWs did not impact the prescribing pattern of FQs [2, 6, 12, 16]. The possible reason for non-adherence to the BWs for FQs may be attributed to the lack of knowledge of the HCPs towards medications with BWs or the content of content of such warnings.

Pakistan is the third highest antibiotic-consuming country among low- and middle-income countries (LMICs) [19]. In Pakistan, FQs are prescribed mostly to patients with GIT infections (bacterial origin) and are among the most commonly prescribed antimicrobials having a prescription percentage of 19.4% in primary health care centers and 22.8% in basic health units [20]. In Pakistan, data suggest that in tertiary care hospitals junior doctors tend to follow the prescriptions of senior or specialist doctors [21, 22], which tends to be the reason for higher prescription of FQs. A study performed among HCPs and prescribers regarding the BWs for FQs this creates an urge to assess the knowledge and a robust system implementation to equip the HCPs with the FDA warning and caution use of these FQs. Despite the FDA alarming BWs of FQs, still the use is high in Pakistan and also self-medication [27] for the management of diseases by general public and antimicrobial resistance [28] is high. This creates a room for health authorities to intervene to tackle that and safe guard the general public from the FQs associated risks.

In our study, 20.4% of the HCPs were prescribers, of which, 79.6% HCPs knowledge score was inappropriate (< 50%). Our findings are consistent with findings of other studies showing inadequate knowledge of HCPs toward BW [10, 29]. Our study findings showed that for inter-professional comparisons showed that knowledge score for indication and adverse effects, the dentists and physicians score was significantly higher than pharmacists and nurses, respectively. Another study performed in Saudi Arabia, revealed different findings in which physicians and pharmacists had better knowledge [10]. The urinary tract infections and respiratory tract infections were reported by majority of the HCPs as an indication for FQs, followed by typhoid fever, otitis externa and skin & soft tissue infections. However, a study in Saudi Arabia showed different findings, i.e., bacterial eye infection, urinary tract infections, and typhoid fever were the most commonly reported indications by HCPs for FQs [10]. Regarding the adverse effects, majority of HCPs in our study reported that photosensitivity or skin reactions, arthropathy and diarrhea were the most frequent adverse effects of FQs. However, literature and findings reported cardiovascular, central nervous system, gastro-intestinal, musculoskeletal and dermatology related adverse effects are mostly linked to different associated adverse effects affecting different systems of the body due to use of FQs [7, 30–33]. Regarding the BW of FQs, majority of HCPs reported mental health side effects, followed by hypoglycemia, peripheral neuropathy and disabling side effects of the tendons, muscles, nerves and joints as the frequent BWs of FQs. This is consistent with the findings of a study on the use of FQs in diabetic patients, which found hypoglycemia with levofloxacin, ciprofloxacin, or moxifloxacin [34]. Furthermore, patients taking moxifloxacin have a higher risk of hypoglycemia than those taking ciprofloxacin [35]. Cardiovascular events including aortic dissection, tendon rupture were associated with the use of FQs [36]. In our study, only 26.9% of the HCPs reported aortic aneurysm/raptures or tears in aorta as BW issued by FDA while 62.9% were not sure about this important BW for FQs. Due to scarcity of specific studies performed in HCPs regarding BW of FQs, it is difficult for us to compare the findings with multiple studies of similar nature; however, a study performed among pharmacist for BW showed insufficient knowledge of pharmacists toward BWs [37] and another study among physicians showed similar findings having inadequate knowledge of BWs [29].

Our study indicated that dentists and physicians possessed higher knowledge score compared to pharmacists and nurses. Likewise, HCPs employed in government sector hospitals had higher knowledge scores compared to those working in private sector hospitals, and community and hospital pharmacies. These findings are inconsistent with the findings of a similar study [10]. Moreover, HCPs in primary and secondary care hospitals demonstrated superior knowledge when compared to their counterparts in tertiary care hospitals and community pharmacies. These disparities might be attributed to the time constraints faced by HCPs in tertiary care hospitals and community pharmacies, resulting from a large patient volume and workload, which limited their ability to stay up-to-date with the latest medication safety information, including BWs. Conversely, professionals in primary and secondary care hospitals generally had more available time for ongoing education and keeping themselves informed about such warnings. In this study, we discovered that HCPs knowledge and understanding of indication, side effects, and BWs was inappropriate.

### Recommendations and practical implications

Based on the study findings, the following recommendations are proposed to address the implications of BWs of FQs on clinical practice. Firstly, regular targeted training sessions, in collaboration with regulatory bodies, should be implemented. This is crucial for the timely dissemination of BWs and safety updates, particularly for medications that are frequently used. Without this, HCPs limited and potentially inappropriate knowledge of FDA-issued BWs for FQs could increase the risk of various medical complications associated with the use of FQs.

Additionally, standardized guidelines specifically for medication safety, especially for drugs with BWs, need to be developed and promoted. Awareness campaigns targeting both HCPs and the public are essential to underscore the importance of medication safety.

Finally, it is imperative for regulatory bodies to ensure the establishment of pharmacovigilance centers across all hospital settings and pharmacies. This will promote the safe use of FQs and enhance overall medication safety.

### Strength and limitation of study

This study's primary strength lies in its novelty; it is the first of its kind conducted among HCPs in the Khyber Pakhtunkhwa area of Pakistan, focusing on their awareness of indications, adverse effects, and the BWs issued by the FDA in 2008. Another significant strength is the study's potential to inform the Ministry of Health and policymakers in developing initiatives to enhance HCPs' knowledge about BWs for FQs and other antimicrobials, thereby safeguarding public health in Pakistan.

However, the study has certain limitations. The scope being restricted to the Khyber Pakhtunkhwa province means the findings may not be generalizable to all HCPs across Pakistan. Moreover, the uneven representation of dentists and physicians compared to pharmacists and nurses suggests a need for larger-scale studies involving a more balanced distribution of these professionals.

Another limitation is the absence of a formal sample size calculation; the study employed a convenience sampling technique, which may affect the robustness of the results. Additionally, there's a potential bias in the responses of HCPs. Given their concern for professional reputation and self-esteem, some might have been reluctant to answer questions about their familiarity with FQs or BWs honestly, potentially skewing the accuracy of the assessment of HCP knowledge regarding BWs for FQs. Efforts were made to maintain the confidentiality of HCP responses to mitigate this issue.

## Conclusion

The findings of this study indicated that HCPs lack sufficient knowledge regarding the appropriate use, safety, and FDA-issued BWs associated with FQs, which could risk the lives of patients and the general public. Of the HCPs, dentists and physicians exhibited a commendable understanding of FQs in these aspects, whereas pharmacists and nurses displayed comparatively lower knowledge levels. Based on the findings of this study, it is essential to implement seminars and lectures aimed at continuously updating the knowledge of HCPs. Furthermore, the study emphasizes the urgent need for a national antimicrobial stewardship program, which would effectively restrict the misuse of antimicrobial particularly FQs by the general public through self-medication and encourage prescribers to promote the safe utilization of these drugs, ultimately safeguarding the health of the Pakistani population.

## Data Availability

The datasets are available from the corresponding author upon reasonable request.

## Abbreviations

FQs: Fluoroquinolones
BW: Box warnings
FDA: Food and Drug Administration
HCPs: Health care professionals
AEs: Adverse events
CNS: Central nervous system
DDDs: Daily defined doses
